# Real-Time Visualization of Joint Cavitation

**DOI:** 10.1371/journal.pone.0119470

**Published:** 2015-04-15

**Authors:** Gregory N. Kawchuk, Jerome Fryer, Jacob L. Jaremko, Hongbo Zeng, Lindsay Rowe, Richard Thompson

**Affiliations:** 1 Department of Physical Therapy, Faculty of Rehabilitation Medicine, University of Alberta, Edmonton, Alberta, Canada; 2 Private Practice, Nanaimo, British Columbia, Canada; 3 Department of Radiology and Diagnostic Imaging, Faculty of Medicine and Dentistry, University of Alberta, Edmonton, Alberta, Canada; 4 Department of Chemical and Materials Engineering, Faculty of Engineering, University of Alberta, Edmonton, Alberta, Canada; 5 School of Medicine and Public Health, University of Newcastle, Callaghan, New South Wales, Australia; 6 Department of Biomedical Engineering, Faculty of Medicine and Dentistry, University of Alberta, Edmonton, Alberta, Canada; University of Nebraska Medical Center, UNITED STATES

## Abstract

Cracking sounds emitted from human synovial joints have been attributed historically to the sudden collapse of a cavitation bubble formed as articular surfaces are separated. Unfortunately, bubble collapse as the source of joint cracking is inconsistent with many physical phenomena that define the joint cracking phenomenon. Here we present direct evidence from real-time magnetic resonance imaging that the mechanism of joint cracking is related to cavity formation rather than bubble collapse. In this study, ten metacarpophalangeal joints were studied by inserting the finger of interest into a flexible tube tightened around a length of cable used to provide long-axis traction. Before and after traction, static 3D T1-weighted magnetic resonance images were acquired. During traction, rapid cine magnetic resonance images were obtained from the joint midline at a rate of 3.2 frames per second until the cracking event occurred. As traction forces increased, real-time cine magnetic resonance imaging demonstrated rapid cavity inception at the time of joint separation and sound production after which the resulting cavity remained visible. Our results offer direct experimental evidence that joint cracking is associated with cavity inception rather than collapse of a pre-existing bubble. These observations are consistent with tribonucleation, a known process where opposing surfaces resist separation until a critical point where they then separate rapidly creating sustained gas cavities. Observed previously *in vitro*, this is the first *in-vivo* macroscopic demonstration of tribonucleation and as such, provides a new theoretical framework to investigate health outcomes associated with joint cracking.

## Introduction

### Background

Sounds emitted from human synovial joints vary in their origin. Joint sounds that occur repeatedly with ongoing joint motion arise typically when anatomic structures rub past one another. In contrast, “cracking” sounds require time to pass before they can be repeated despite ongoing joint motion. Although various hypotheses have been proposed over many decades regarding the origin of cracking sounds, none have been validated; the underlying mechanism of cracking sounds remains unknown.

### History

In 1947, Roston and Wheeler Haines [1] published the first scientific study toward describing the origins of joint cracking. Their experiment used serial radiography to visualize joint cracking when distraction forces were applied to metacarpophalangeal (MCP) joints. Their results characterized the sequence of gross articular events that define joint cracking. The process begins with the resting phase where joint surfaces are in close contact. In this stage, a light distraction force will barely separate the joint surfaces. With a greater distraction force, the surfaces resist separation until a critical point after which they separate rapidly. It is during this rapid separation phase that the characteristic cracking sound is produced. Following cracking, the joint is in a refractory phase where no further cracking can occur until time has passed (approximately 20 minutes). Importantly, post-cracking distraction also reveals the presence of a “clear space” assumed by Roston and Wheeler Haines to be a vapour cavity. This cavity, described by some as a bubble, has been thought to form as distraction forces decrease pressure within the synovial fluid to the point were dissolved gas comes out of solution. Importantly, Roston and Wheeler Haines linked the production of the cracking sound to the formation of this clear space, a phenomenon first described in 1911 [2] but thought by some to occur only in unhealthy joints [3] until demonstrated to also occur in normal joints[4].

This interpretation of joint cracking stood as the standard for 24 years until 1971 when Unsworth, Dowson and Wright [5] refuted this view by stating that the exact mechanism of joint cracking “was in doubt”. Although Unsworth et al. used a similar radiographic procedure to confirm the same sequence of events described by Roston and Wheeler Haines, they arrived at a different conclusion. Specifically, Unsworth et al. speculated that the formation of a clear space, or bubble, was not the source of joint cracking, but rather cracking was caused by the subsequent collapse of the bubble. This idea was likely influenced by the realization that bubble collapse could cause damage in surfaces adjacent to the bubble itself [6]. First described by Rayleigh in 1917 [7], cavitation collapse came into the fore in the late 1960s as a source of significant damage in marine equipment [6] such as propellers, hydrofoils [8].

As a result, publications since 1971 have referenced Roston [9–11] or Unsworth [12–24] or both [5,11,25–39] when describing joint cracking. Adding to the confusion, others [25] have suggested that sound produced during joint cracking occurs through ligamentous recoil. Still others [18,19,25,26] advocate for an additional mechanism known as viscous adhesion or tribonucleation [40,41], a process that occurs when two closely opposed surfaces are separated by a thin film of viscous liquid. When these surfaces are distracted, viscous adhesion or tension between the surfaces resist their separation. Then, as distraction forces overcome the adhesive forces, the surfaces separate rapidly creating a negative pressure. This negative pressure, combined with the speed with which the surfaces separate, can create a vapour cavity within fluid much like a solid that has been fractured [42–44].

Unfortunately, no direct evidence exists to resolve these differing perspectives regarding the mechanism of joint cracking. While many have used various radiographic means to record events associated with joint cracking [1,5,10,45], these techniques have a number of limitations which conspire to obscure intra-articular events due to low space-time resolution, insufficient contrast and superimposition of structures.

Given the above, the objective of this study was to characterize the events associated with joint cracking within the joint itself using real-time cine magnetic resonance imaging (cine MRI). Here we present direct evidence from cine MRI that the mechanism of joint cracking is related to cavity formation rather than bubble collapse.

## Materials and Methods

### Ethics Statement

An adult male subject possessing the ability to crack his MCP joints provided full informed, written consent to participate in this study approved by the Human Ethics Research Board of the University of Alberta.

### Preparation

**Fig 1:**
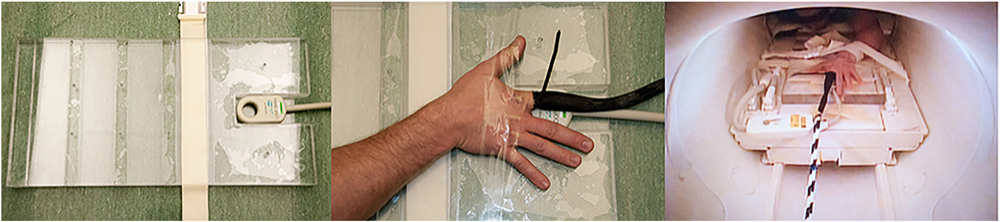
The radiofrequency coil inside the clear housing (left). The metocarpophaangeal (MCP) joint of interest centred over the bore of the radiofrequency coil (middle). The participant’s hand within the imaging magnet (right).

Ten MCP joints from a single participant were studied over two sessions with one finger at a time isolated for imaging. With the subject prone on the imaging gantry, the finger of interest was inserted into a tubular finger trap [46] that covered the finger from the apex to midway between the MCP and the proximal interphalangeal joint (Fig. 1). This end of the tube was tightened to the finger with a releasable tie. The opposite end of the tube was connected in-series to a ¼” diameter cable. The MCP of interest was then centered over top of a radiofrequency coil designed for MRI imaging of digits with the long axis of the finger perpendicular to the coil bore (Fig. 1).

### Imaging

Imaging studies were performed on a Siemens Sonata 1.5T system (Sonata; Siemens Healthcare; Erlangen, Germany) using the provided Siemens finger coil. Before and after MCP distraction, static magnetic resonance images were obtained of the MCP joint (3D T1 weighted GRE: Field of view = 160 x 120 mm, 256 x 192 matrix, 2 mm slice thickness, Flip angle = 30 degrees, TR = 20.0 ms, TE = 3.17 ms, bandwidth = 250 Hz/pixel). During distraction of the MCP joint, cine MRI was acquired from the midline of the joint at a rate of 3.2 frames per second until the distraction force was removed following the cracking event. Cine imaging parameters for a single shot steady-state free-precession (SSFP) pulse sequence were as follows: Field of view = 200 x 75 mm, 192 x 72 matrix, 5 mm slice thickness, Flip angle = 70 degrees, TR = 4.30 ms, TE = 2.15 ms, bandwidth = 1000 Hz/pixel.

### Joint Distraction

With the subject prone, the hand and radiofrequency coil were secured to the imaging gantry then positioned in the magnet (Fig. 1). The cable attached to the finger of interest was then threaded through the magnet so that it exited on the side opposite the subject. During cine MRI acquisition, a slowly increasing distraction force was applied manually through the cable until the subject indicated the occurrence of joint cracking. At any time, the subject could request the process be stopped for any reason (which did not occur). In 5 MCP joints, distraction was ceased immediately after the cracking event. In the remaining 5 cases, distraction forces were maintained for approximately 5 seconds after cracking.

#### Image Analysis

Static images were displayed with software supplied by the magnet manufacturer. Cine MRI images were loaded as imaging sequences into ImageJ software [47] for further analysis. Within this software, images prior to the start of distraction and after the cessation of distraction were deleted from the imaging sequence. The remaining image sequence was then converted into binary images using default threshold settings within Image J. The space between the joint surfaces was then measured prior to joint distraction, immediately after the cracking event (the frame immediately following rapid joint separation) and once distraction forces were ceased. Measurement of joint space separation was performed by a custom Image J script that converted the images in the cine sequence to a binary format. In each cine frame, joint edges were detected automatically through thresholding and the total space between joint surfaces measured within a defined region of interest. In addition, MRI signal intensity was evaluated as a function of time in the region of interest where cavity formation occurred as well as in control areas where signal intensity was not expected to change (i.e. cancellous bone). All images were reviewed by an imaging physicist and two certified radiologists using native contrast settings.

## Results

All ten MCP joints imaged resulted in joint cracking as confirmed by the subject and the cable operator.

**Fig 2:**
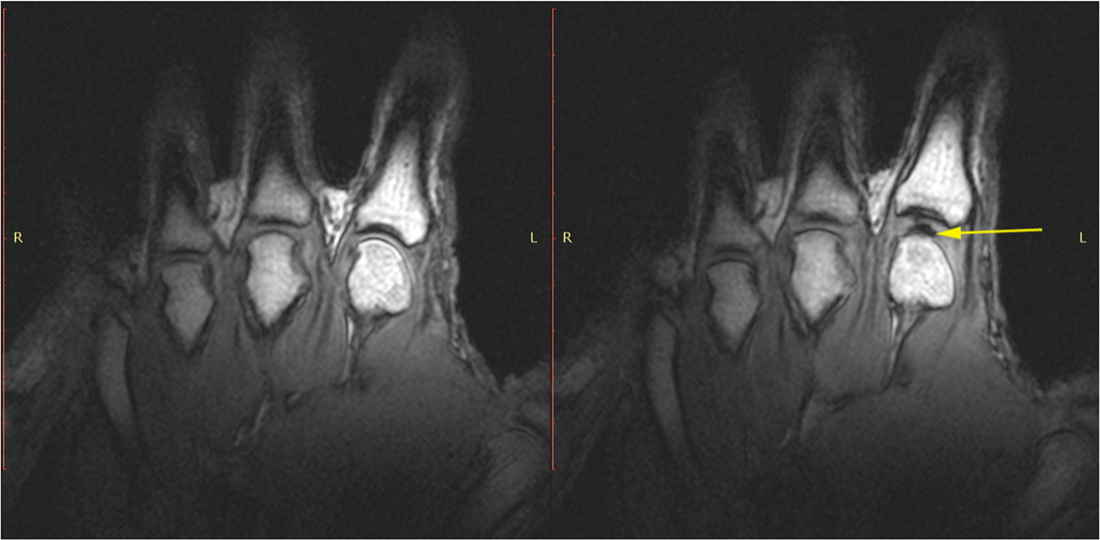
T1 static images of the hand in the resting phase before cracking (left). The same hand following cracking with the addition of a post-cracking distraction force (right). Note the dark, interarticular void (yellow arrow).

Static images revealed normal MCP joints with the expected lack of any gaseous cavity prior to joint distraction (Fig. 2). Following the cracking event, static imaging with the addition of MCP distraction yielded a dark intra-articular void (Fig. 2).

**Fig 3:**
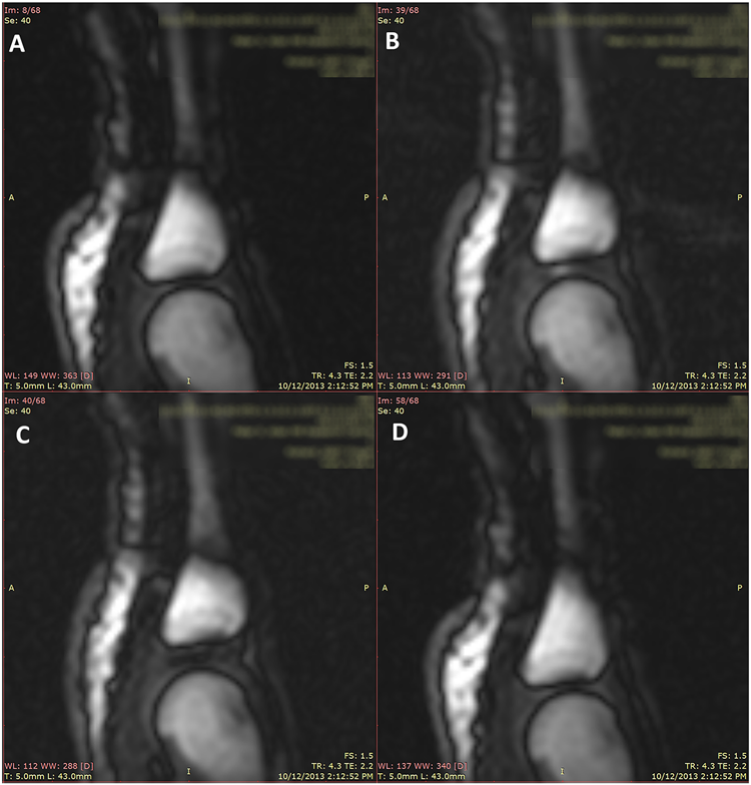
Still frames from a representative trial of joint cracking in the same MCP joint. The right 4^th^ MCP joint in the resting phase (A). The MCP joint as seen during distraction of the MCP joint in the frame just prior to joint cracking / joint separation (B). The MCP joint visualized in the next frame immediately after joint cracking (C). The joint in the refractory phase immediately after removal of distraction forces (D).

**Fig 4:**
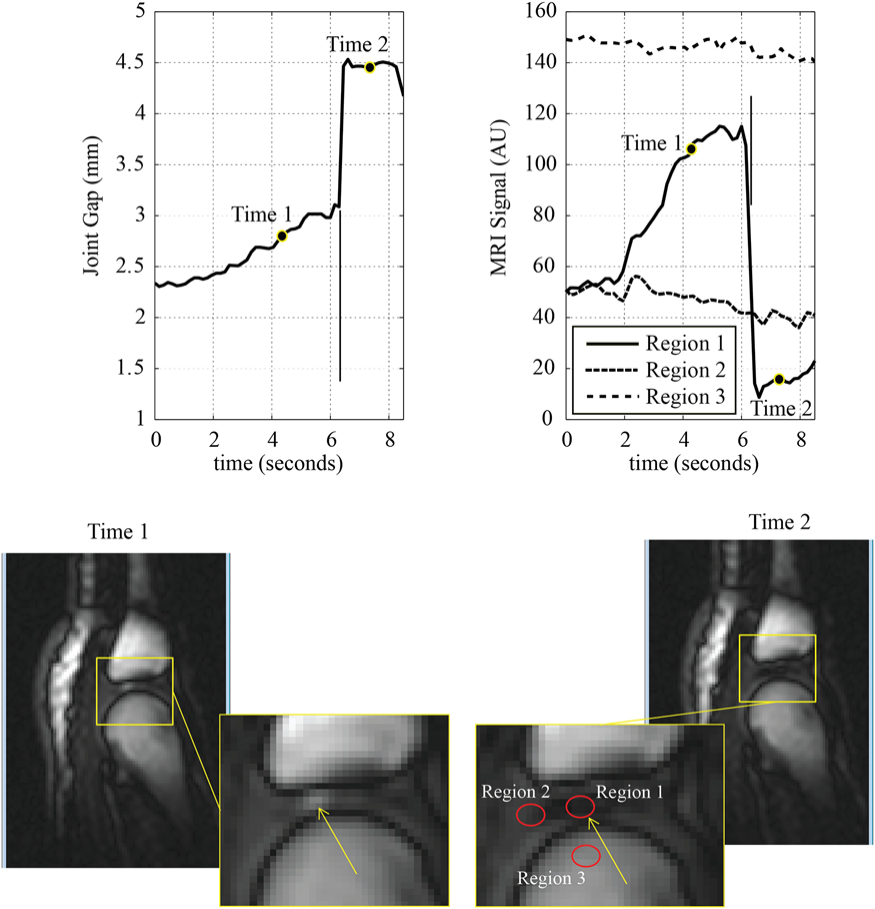
Time series plots for joint separation distance and signal intensity over the course of a representative MCP joint cracking (plots). Cine MRI images displayed are those immediately prior to, and after, joint cracking with zoomed regions to demonstrate areas where signal intensities were measured for the region of interest as well as control regions.

Cine MRI imaging revealed a sequence of events consistent with that outlined by Roston and Wheeler Haines [1]. A video of these events can be viewed in the supplemental material (S1 Video). Four still frames from the 4^th^ right MCP joint depicting the characteristic intra-articular events associated with joint cracking are presented in Fig. 3: resting joint geometry (Fig. 3A), a time frame just prior to cracking (Fig. 3B), a time frame just after cracking (Fig. 3C) and a final frame following release of distraction forces (Fig. 3D). In the supplemental materials, a series of images is presented showing the moment just after joint cracking in all MCP joints investigated (S1 Fig.). Fig. 4 shows a time series of these events to display joint separation distance and changes in MRI signal-intensities for a representative finger cracking event. The joint separation distance shows a slow increase to the point of joint release at 6.2 seconds, as indicated by the vertical marker (left frame). The MRI signal intensity within the intra-articular space (Region 1) drops to reveal a signal void at the same time as the joint expands (6.2 seconds). Control regions in the fluid outside of the intra-articular space (Region 2) and in the bone (Region 3) show relatively unchanging signal intensities over the experiment. All regions were moved in each frame to track the motion of the bones. Finally, the signal intensity in the intra-articular space (Region 1) showed a steady increase with distraction just prior to joint cracking (Fig. 3B). Still images in the bottom of Fig. 4 highlight frames prior to, and just after, joint cracking which demonstrate a signal increase in Region 1 and the subsequent signal drop in the same region.

Joint cracking always occurred over a single imaging frame which meant its duration was less than the duration of single frame (i.e. 310 ms). Wilcoxon Signed-ranks testing showed a significant difference in joint space in the cine frame prior to (0.93 mm +/- 0.73 mm STD) and after (1.89 mm +/- 0.59 mm STD) rapid surface separation/cracking (p = 0.001). The mean joint separation space prior to testing and after testing was not significantly different (p = 0.21).

The signal void as a result of joint cracking was observed in all 10 MCP joints studied and varied in size, shape and location. When distraction forces were maintained following joint cracking, the black void remained then disappeared from the field of view typically when distraction forces were removed and the joint surfaces allowed to re-approximate (Fig. 3D).

## Discussion

This study employed cine MRI to visualize joint cracking in real time. To our knowledge, cine MRI has not been used previously to characterize this phenomenon. Congruent with historic results, cine MRI demonstrated minimal joint surface separation in the resting phase prior to joint cracking followed by rapid joint separation during the crack itself. Incongruent with the prevailing perspective from the last half century, cine MRI revealed rapid cavity inception associated with concurrent sound production and joint separation. Following these events, the resulting cavity was never seen to collapse; the cavity formed at the time of rapid joint separation then persisted past the point of sound production.

Dark signal intensities in the joint immediately following cracking on both cine MRI imaging (a balanced SSFP pulse sequence with a characteristic mixed (T_2_/T_1_) weighting) as well as in the higher resolution T_1_ weighted static images, supports the presence of an air region of interest. Specifically, a significant and rapid increase in the fluid T_1_ values, which could reduce the signal intensity in both of these acquisitions, is implausible, and thus the reduction in signal is most likely due to a reduction in spin density associated with the formation of an air space. The gradual increase in signal intensity in the same region just prior to the cracking is suggestive of fluid accumulation during this phase of the finger cracking.

### Events consistent with tribonucleation

Our results offer direct experimental evidence that joint cracking is the result of cavity inception within synovial fluid rather than collapse of a pre-existing bubble. These observations are consistent with tribonucleation, a known process where opposing surfaces resist separation until a critical point where they separate rapidly resulting in vapor cavities that do not collapse instantaneously.

Specifically, tribonucleation explains each phase of the joint cracking sequence described originally by Roston and Wheeler Haines [1]. The resting phase where distraction forces result in minimal joint separation is explained by viscous attraction between joint surfaces. With sufficient distraction force, those adhesive forces are overcome which explains the rapid separation of joint surfaces. The resulting drop in synovial pressure allows dissolved gas to come out of solution which explains the “clear space” (a.k.a. bubble, cavity, void, fluid fracture) created within the joint. This cavity persists past the point of sound production; a subsequent collapse is never visualized. Importantly, the cavity does disappear from the region of interest with subsequent cessation of distraction forces, but well after joint cracking has occurred.

### Interpretation of prior studies

Our results are consistent with those of Roston and Wheeler Haines [1]. Their classic study using serial radiographs correctly identified the sequence of events that characterizes joint cracking. Although technical limitations did not allow them to see formation of the cavity during sound production, but only its presence after its formation, they correctly identified creation of the clear space as the defining event of joint cracking. Furthermore, many of their speculations were consistent with tribonucleation. These included prophetic comments that the 1) distraction force must be applied to overcome tension within the synovial fluid (not within the soft tissues) before cracking can occur and that 2) the inherent tension forces that kept the joint surfaces together add stability to the joint itself.

Alternatively, the suggestion by Unsworth et al. [5] that joint cracking was the result of cavity collapse, is a sensible one given the tremendous amount of work at the same time that defined bubble collapse to be a source of damage in marine equipment. While the 1971 paper from Unsworth et al. [5] made significant contributions in terms of the role of joint symmetry in joint cracking, composition of synovial gases and providing an explanation for the refractory period, they did not provide any direct evidence of a cavity collapse despite their conclusion. Given that the cavity which forms after joint cracking disappears from view when distraction forces are removed, but then appears again with additional distraction, Unsworth et al. [5] may have mistaken this disappearance for bubble collapse. Even if the “bubble” is reabsorbed after joint cracking to then be reformed in some fashion with subsequent distraction, the appearance and persistence of a cavity following rapid joint separation does not support bubble collapse as a mechanism of joint cracking. We also observed that the joint space before and after testing did not change significantly. This finding suggests that the resting joint orientation is not changed by the cracking event in the MCP. This is in disagreement with Unsworth et al. [5] who suggested that resting MCP joint space increases following cracking.

While our work provides new insights into defining the mechanism underlying joint cracking, this new visualization technique opens novel avenues for investigation. Specifically, cine MRI revealed a new phenomenon preceding joint cracking; a transient bright signal in the intra-articular space. While not likely visualized gas given the imaging parameters employed, we do not have direct evidence to explain this observation. We speculate this phenomenon may be related to changes in fluid organization between cartilaginous joint surfaces and specifically may result from evacuation of fluid out of the joint cartilage with increasing tension. If so, this sign may be indicative of cartilage health and therefore provide a non-invasive means of characterizing joint status.

### Limitations

The slice thickness used for cine MRI prevented us from visualizing the joint in its entirety. As such, it was not possible to see what happened within all regions of the joint during cracking. Future studies that image peripheral areas of the MCP may reveal the fate of the cavity formed after rapid joint separation which does not collapse at the time of joint cracking, but disappears from the region of interest when distraction forces on the joint are removed. The current slice thickness in cine MRI cannot establish if the cavity formed after joint cracking migrates to the peripheral region of the joint or is resorbed when distraction forces cease. Similarly, when distraction forces are provided in the refractory phase, our data does not assist us in determining if the observed cavity reforms from gas nuclei migrating together from the periphery of the joint or if a new cavity is formed *de novo* from solution.

In addition, we presume that rapid joint separation with cavity formation does not occur at the same traction force in each finger. Unfortunately, traction forces were not measured in this experiment due to incompatibility of available force measuring equipment with MRI..

Last, this work does not explain the magnitude of the sound caused by cavity formation. Although some have noted the production of sound during cavity formation through tribonucleation [48–50], the amplitude of the generated sound from these experiments would appear to be small whereas joint cracking can easily be heard across a room. Given the above, our *in vivo* results may be the largest example of tribonucleation and subsequent sound production observed to date.

## Conclusions

Our data support the view that tribonucleation is the process which governs joint cracking. This process is characterized by rapid separation of surfaces with subsequent cavity formation, not bubble collapse as has been the prevailing viewpoint for more than a half century. Observed previously *in vitro*, this work provides the first *in-vivo* demonstration of tribonucleation on a macroscopic scale and as such, provides a new theoretical framework to investigate health outcomes associated with joint cracking. This framework will allow scientists to compare and contrast this process against tribonucleation observed between inanimate surfaces, an approach that may reveal how joint cracking affects cartilaginous joint surfaces. Presently, the literature in this area is confusing in that the energy produced during joint cracking is though to exceed the threshold for damage[51], but habitual knuckle cracking has not been shown to increase joint degeneration [52]. Ultimately, by defining the process underlying joint cracking, its therapeutic benefits, or possible harms, may be better understood.

## Supporting Information

S1 Fig Still cine MRI frames from each of the 10 metocarpophalangeal joint investigated in this study. (TIF)

S1 Video Real-time cine magnetic resonance imaging of the human metacarpalphalangeal joint undergoing traction. Note that the joint surfaces stay in close contact. Then, as traction forces increase, a focal area of increased signal intensity is visualized prior to the critical point where rapid joint separation occurs and cavity formation is then visualized.
(MP4)
