# Supplementary figures and images for: Real-Time Visualization of Joint Cavitation
Source: PLoS One. 2015 Apr 15;10(4):e0119470. doi: 10.1371/journal.pone.0119470 (PMC4398549; doi:10.1371/journal.pone.0119470)

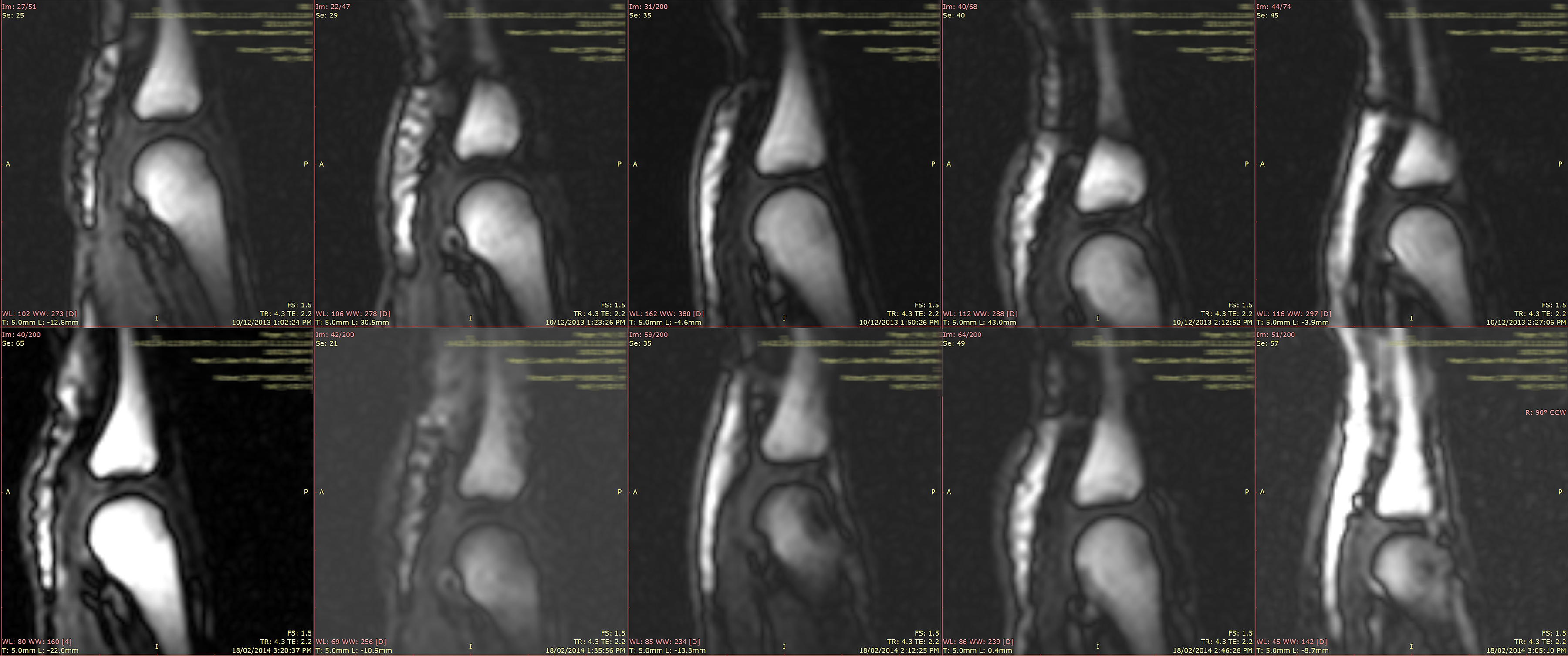

Supplement: S1 Fig — (TIF) [file pone.0119470.s001.tif]
